# A Case Series of Persistent SARS-CoV-2 Infection in Immunocompromised Pediatric Patients

**DOI:** 10.1155/2023/1699770

**Published:** 2023-05-16

**Authors:** Mohamed Y. Ahmed, Jane B. Taylor, Rajesh K. Aneja, Qian Wang, John V. Williams

**Affiliations:** ^1^Department of Pediatrics, Division of Pediatric Critical Care Medicine, Indiana University School of Medicine, USA; ^2^Department of Pediatrics, Division of Pulmonology, University of Pittsburgh School of Medicine, UPMC Children's Hospital of Pittsburgh, USA; ^3^Department of Critical Care Medicine, University of Pittsburgh School of Medicine, USA; ^4^Department of Pathology, University of Pittsburgh School of Medicine, USA; ^5^Division of Infectious Diseases, University of Pittsburgh School of Medicine, UPMC Children's Hospital of Pittsburgh, USA

## Abstract

Diagnosis and management of SARS-CoV-2 infection in immunocompromised patients are extremely challenging. These patients can have atypical clinical courses, and there is a paucity of data regarding clinical features, diagnostic findings, and the safety and efficacy of available therapeutic agents used to treat COVID-19 in these patients. In this case series, we report atypical COVID-19 presentations in 4 immunocompromised pediatric patients who were admitted with acute respiratory failure after an initial diagnosis of COVID-19 a few weeks earlier. All patients included in this cohort showed persistent worsening respiratory symptoms for several weeks before hospital presentation. While they manifested common COVID-19 sequelae, they also had rare COVID-19-related pathognomonic and radiographic features developed along their hospital course. Multiple therapeutic agents were used in their COVID-19 management, including corticosteroids, remdesivir, and monoclonal antibodies. All three patients who have received concurrent therapy with remdesivir, hydrocortisone, and monoclonal antibodies survived, and only one patient died as a direct complication of COVID-19 ARDS with secondary pulmonary mucormycosis. Our outcomes suggest the potential benefit of remdesivir use in combination with hydrocortisone and monoclonal antibodies in the management of severe COVID-19 ARDS in this group, as well as the importance of close surveillance and early administration of broad empirical antimicrobial and antifungal coverage if clinically indicated in this high-risk population.

## 1. Introduction

Typically, immunocompetent patients who are acutely infected with SARS-CoV-2 will have a wide range of presentation, from asymptomatic, to mild, moderate, or severe symptoms. This variation tends to be associated with other comorbidities, such as obesity or underlying lung disease. Reported persistent symptoms in this group include mild respiratory symptoms (cough and chest pain) or other constitutional or behavioral symptoms, which can last for several weeks to months [1–4]. Diagnosis and management of SARS-CoV-2 infection in immunocompromised patients are extremely challenging due to the paucity of data regarding the clinical course, features, diagnostic findings, as well as safety and efficacy of available therapeutic agents used to treat COVID-19 in these patients. In this case series, we report atypical COVID-19 presentations in 4 immunocompromised pediatric patients who were admitted with acute respiratory failure after an initial diagnosis of COVID-19 a few weeks earlier.

### 1.1. Case 1

**Figure 1:**
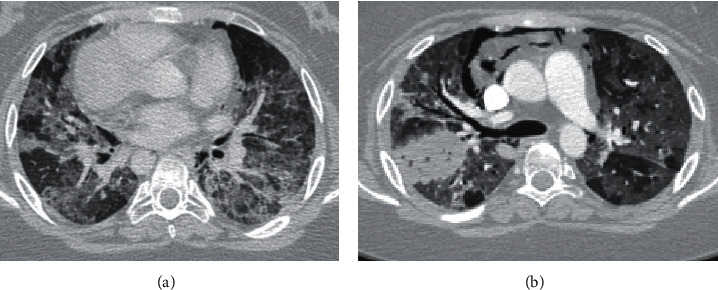
(a) Chest CT scan obtained for case 1, showing diffuse ground glass opacities consistent with COVID-19 pneumonia. (b) Repeat chest CT scan for case 1 5 days later, showing a new right-sided pulmonary infarct, found later to be secondary to mucormycosis angioinvasive fungal infection.

Patient 1 was a 16-year-old female with a history of relapsing B-cell ALL who was status post (s/p) allogeneic HLA-mismatched unrelated donor peripheral stem cell transplant. She required chronic immunosuppression. Six months before COVID diagnosis, she developed biopsy-confirmed graft versus host disease (GVHD) in the gastrointestinal tract. Three months later, she developed mixed restrictive and obstructive lung disease of unclear etiology. During her initial workup, her sputum culture was positive for *Bordetella bronchisepticum*, and she completed a 2-week course of levofloxacin for presumed bacterial bronchitis. Her adenovirus PCR was also positive at a low level (40 copies). There was concern for an infectious process versus bronchiolitis obliterans from adenoviral infection or GVHD. Two months later, she presented with worsening cough and hypoxemia requiring up to 1 L/min of oxygen. At this time, she tested positive for COVID-19. She was admitted and treated with 5 days of IV remdesivir and IV dexamethasone after meeting clinical criteria for age and hypoxemia. She was weaned off supplemental oxygen and discharged 1 week later; however, she continued to have a persistent cough without fevers or dyspnea. Three weeks later she redeveloped dyspnea and was readmitted with hypoxemia. Her repeat COVID-19 PCR testing was positive, and lab-work again showed sputum culture positive for *B. bronchisepticum* and a positive adenovirus PCR (200 copies). She was started on IV cefepime for bacterial bronchitis and per oral (po) cidofovir for acute adenoviremia. She continued *Pneumocystis jirovecii* (PJP) prophylaxis. COVID-19-targeted therapeutics were deferred at that time. Her symptoms continued to worsen, and she was transferred to the pediatric intensive unit (PICU) for initiation of noninvasive ventilatory support (NIV). Labs showed progressive anemia, thrombocytopenia, uremia, and elevated D-dimer. CT-angiogram (Figure 1(a)) showed diffuse ground glass opacities without pulmonary emboli. Antibiotic therapy was broadened to include vancomycin for MRSA coverage, and she continued cefepime. The constellation of anemia, thrombocytopenia, and uremia was suggestive for thrombotic microangiopathy (TMA), with atypical hemolytic-uremic syndrome (aHUS).

**Figure 2:**
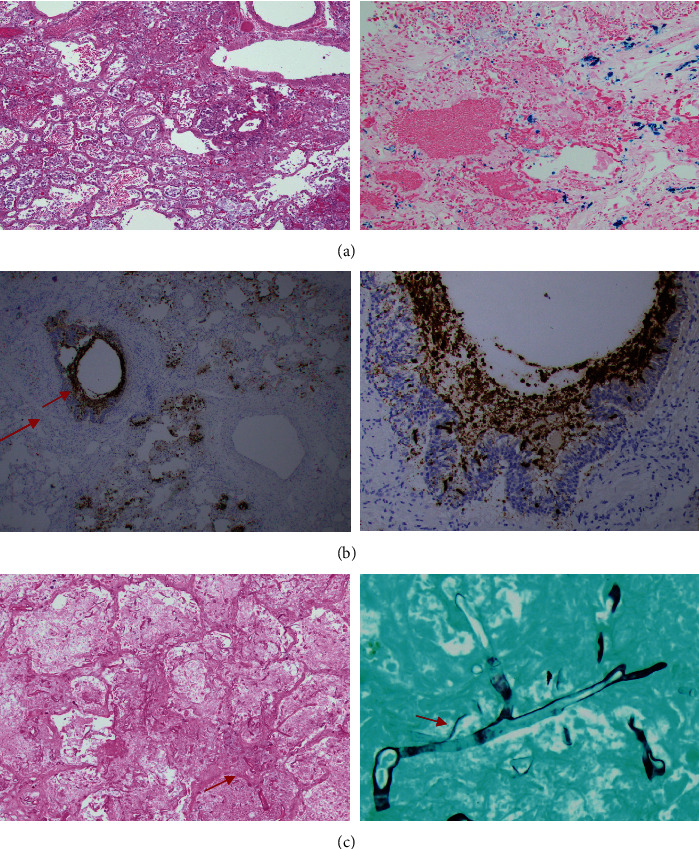
(a) Lung autopsy from case 1 showing diffuse alveolar hemorrhage. (b) Lung autopsy from case 1 showing positive in situ hybridization for COVID-19 within the alveolar macrophages and alveolar epithelium, suggestive for an acute COVID-19 pneumonia/infection. (c) Lung autopsy obtained from case 1, showing fungal (*Mucor*) elements in the right upper lobe, with the classic branching pattern, confirmed by GMS and tissue culture.

It was unclear if the hyperinflammatory response was triggered by active COVID-19 infection, sequelae of sepsis, or adenoviremia. Accordingly, she received eculizumab for management of aHUS, with a 7-day course of IV dexamethasone. Her clinical course continued to deteriorate, and she was intubated and placed on extracorporeal membrane oxygenation (ECMO) support. A right upper lobe opacity was noted on her chest radiograph and CT (Figure 1(b)) with progressive pneumomediastinum, concerning for lung infarction. At that time, the extensive lung injury was deemed to be irreversible, and with the extremely high risk for perioperative morbidity and mortality with a lung transplant, life support was withdrawn after discussion with her family. Autopsy showed diffuse alveolar damage and multiple foci of alveolar hemorrhage (Figures 2(a) and 2(b)). There was a 5 cm infarcted lesion with an angioinvasive fungal infection (Figure 2(c)). A GMS stain was positive, and a culture grew mucormycosis/*Rhizopus* speciation. COVID-19 in situ hybridization showed positive reactivity in many alveolar macrophages and some respiratory epithelium, suggesting active COVID-19 infection at the time of death.

### 1.2. Case 2

**Figure 3:**
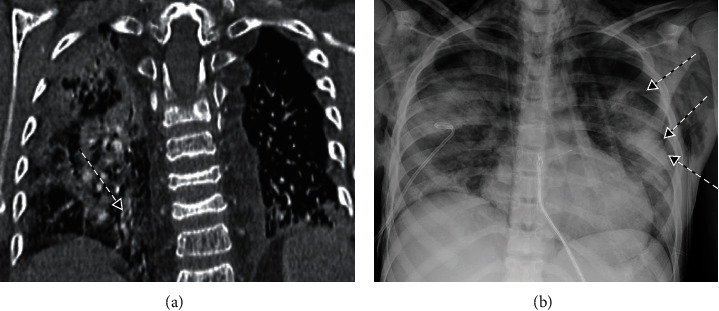
Variable COVID-19-related pathologies in immunocompromised patients: (a) chest CT angiogram demonstrating filling defects in the posterior and basal branches of pulmonary arteries, consistent with pulmonary embolism; (b) AP chest radiograph showing bilateral moderate to large pneumothoraxes.

Case 2 was a 12-year-old female with history of B-cell ALL s/p unrelated donor bone marrow transplant (BMT) in 2015, GVHD, s/p renal transplant in 2021, drug reaction with eosinophilia and systemic symptoms (DRESS), multiple antibiotic allergies, hypogammaglobulinemia requiring monthly IVIG, and chronic lung disease of unclear etiology with left-sided diaphragmatic paresis requiring nocturnal BiPAP at night. In December 2021, she was diagnosed with COVID-19 in the setting of fever without hypoxemia, received monoclonal antibodies in the ER given her high risk for hospitalization with immunosuppression, and was discharged home. Her fever subsided, but she continued to have a persistent dry cough. Two weeks after initial presentation, she became hypoxemic and required supplemental oxygen. Three days later, she was admitted for worsening hypoxemia and fevers. CXR on admission showed worsening left lower lobe opacities. Linezolid was started for presumed bacterial superinfection without any significant improvement, and she was eventually transferred to the ICU for worsening fever and hypoxemia. In the ICU, she was intubated, and broad antimicrobial coverage was started with IV vancomycin, cefepime, and amphotericin. CT angiogram (Figure 3(a)) showed segmental bilateral lung infiltrates and a subsegmental left pulmonary embolism, suggestive for thrombotic microangiopathy (TMA). Bronchoalveolar lavage (BAL) detected a high COVID-19 viral load, suggestive for an active SARS-CoV-2 infection. BAL cytology showed numerous hemosiderin-laden macrophages, concerning for diffuse alveolar hemorrhage (DAH). All other viral PCR panels, bacterial, fungal, and AFB cultures were negative. She was started on a 5-day course of IV remdesivir and SC Lovenox for a pulmonary embolism (PE). She also received IV methylprednisolone, 1 dose of anakinra, and 1 dose of tocilizumab for COVID-19-related TMA/DAH. The patient gradually improved, and repeat tracheal aspirates showed decreasing subsequent viral loads. She was extubated 1 week later, weaned to room air after 10 days, and then discharged home.

### 1.3. Case 3

Case 3 was a 14-year-old female with end stage renal disease (ESRD) secondary to P-ANCA glomerulonephritis requiring peritoneal dialysis. She was diagnosed with COVID-19 in early December 2021 and continued to have persistent fever with cough. Two weeks from onset of symptoms, she was hospitalized for observation and dialysis. At that time, she remained intermittently febrile with mild respiratory symptoms but did not have SOB or hypoxemia. CXR showed bilateral fine reticulonodular infiltrates without any focal consolidation, and she was discharged after 2 days of observation. After discharge, fever and cough persisted. Upon presentation for dialysis, she had fever to 102°F, with rapid breathing. Initial testing showed a positive COVID-19 nasal swab, CXR demonstrating bilateral alveolar infiltrates with small bilateral pleural effusions, lab-work showing significantly elevated inflammatory markers (CRP 24.7, procalcitonin > 200, IL-6 71.5), and downtrending hemoglobin. Broad antimicrobial coverage was started, and she was admitted to the PICU for respiratory support. She was eventually intubated for mechanical ventilation, and a BAL detected high COVID-19 viral load via RT-PCR (detection threshold at cycle 17/45, indicating high viral load), suggestive for active viral disease, so a 10-day course of IV remdesivir was started. She initially received steroids for a possible GPA vasculitis flare with respiratory involvement. As her clinical course progressed, she received multiple immunosuppressive agents including pulse steroids, anakinra, plasmapheresis, and IVIG for possible reactivation of ANCA+ vasculitis and hyperferritinemia (13,098) secondary to COVID-19 ARDS. Her course was complicated with other morbidities including pneumomediastinum and pneumothorax (Figure 3(b)), requiring peritoneal drain and chest tube placement. She showed gradual improvement and was extubated to CPAP with a quick wean to room air. After 4 weeks of admission, chest tubes and peritoneal drains were removed. The patient was discharged home on room air after 5 weeks of admission.

### 1.4. Case 4

Case 4 was a 19-year-old male with granulomatosis with polyangiitis on chronic immunosuppression therapy. He was diagnosed with COVID-19 in April 2021 in the setting of fever and chills, with complete recovery without treatment. Seven days later, he developed fever and productive cough. A CXR showed diffuse bilateral infiltrates, and a CBC showed downtrending hemoglobin. He was diagnosed with a possible GPA flare with suspected DAH. Given his recent recovery from COVID, it was initially thought that COVID-19 pneumonia was unlikely. A repeat nasal swab for COVID-19 was not done since his recent COVID infection would still produce positive results. He was admitted for IV rituximab and IV pulse steroids. He was discharged on a steroid taper. After discharge, his fever subsided, but his breathing and cough worsened. In an urgent outpatient clinic follow-up visit, he was tachypneic and mildly hypoxemic and required readmission. A bronchoscopy was performed, and the BAL was positive for COVID-19, so a 3-day course of IV remdesivir was started. The bacterial culture was positive for *Haemophilus influenzae*, so he was treated with a 10-day course of IV ampicillin/sulbactam and PO amoxicillin. He was discharged after 3 days of admission, after resolution of his symptoms.

## 2. Discussion

Persistent COVID-19 symptoms in immunocompromised patients have been reported. In one immunosuppressed adult patient, severe persistent COVID-19 respiratory symptoms were treated with two 10-day courses of remdesivir starting at 24 and 45 days after fever onset. In that patient, pneumonia and spiking fevers initially remitted but relapsed after discontinuation [5]. Here, we report a similar presentation of prolonged COVID-19 in immunocompromised pediatric patients. All patients, despite their prolonged and/or protracted courses, had various CXR and CT scan radiographic findings that are typically demonstrated in acute COVID-19 infection, including diffuse ground-glass opacities (either due to infection or diffuse alveolar damage) and diffuse bilateral infiltrates, with or without focal consolidations [6–8]. Our patients also showed much less frequent findings associated with COVID-19 infection (Table 1), including spontaneous pneumothorax [7, 9], pulmonary emboli, pneumomediastinum, and lobar infarcts with cavitary lesions secondary to a superimposed mucormycotic fungal infection. All patients had a positive nasal swab PCR for COVID-19. Establishing the diagnosis of active persistent COVID-19 infection was challenging in this group of patients given their prolonged duration of symptoms, and known persistence of COVID + testing in immunosuppressed individuals with nonactive viral products after the active replication cycle has passed. Their ongoing clinical symptoms were shown to be secondary to sustained viral replication and inability of the immune system to clear the infection in a typical amount of time expected for a viral infection. Obtaining a BAL to demonstrate high COVID-19 viral load was helpful in differentiating between active infection and persistent viral shedding. However, based on our data, obtaining a BAL may not be essential for clinical diagnosis if other suggestive clinical and radiographic features are present.

Patients with severe clinical presentations showed various features associated with known severe COVID-19 infection, including complement-mediated ARDS, thrombotic microangiopathy, diffuse alveolar hemorrhage [10–12], and other complement-mediated multiorgan dysfunction syndrome (MODS) which includes atypical hemolytic-uremic syndrome (aHUS) [13]. Superimposed fungal infections with *Aspergillus* and mucormycosis have also been reported in COVID-19 patients [14, 15] and associated with worse clinical outcomes and mortality. There have been numerous reports showing that patients with hematological malignancy, stem cell, and solid organ transplants and patients on chronic steroids are at higher risk of developing secondary fungal infections [16, 17]. A few reports suggested that SARS-CoV-2 infection alone might alter the immune system by affecting T lymphocytes, particularly CD4+ and CD8+ T cells, leading to an overall reduction in the number of lymphocytes and T cells [18, 19]. Lung autopsy in the fatal case we report showed evidence of active COVID-19 infection, and fungal elements were detected in the right lung infarction. Additionally, routine antifungal prophylaxis did not prevent the mycotic infection in our first case. Our data suggests the importance of maintaining a high clinical suspicion for secondary bacterial and fungal infections in this high-risk population and initiating broad antimicrobial and antifungal coverage if rapidly deteriorating. In addition, there is a paucity of data with regards to the ideal therapeutic agents that can be provided.

Corticosteroids were used in all severely ill patients who required admission to the ICU. There has been some clinical variation in the use of remdesivir in immunosuppressed patients, particularly because of the prolonged duration between the onset of symptoms leading to hospitalization. The timeframe presented in our four cases was outside the normal window of treatment, which initially made it challenging to establish the diagnosis of active COVID-19 infection prior to obtaining the BAL with high viral loads. This made it challenging to predict if there is any benefit of remdesivir use to prevent mortality. In contrast to the single adult case report, remdesivir appeared to be beneficial in 3 of the pediatric patients who received it within the initial 3-5 days of PICU admission as evidenced by the reduction of active viral loads on serial BAL cultures and tracheal aspirates. Other therapeutic interventions that were used were monoclonal antibodies, tocilizumab, anakinra, and eculizumab. The decision to choose each agent was on a case-by-case basis, depending on the underlying cause of immunosuppression and other associated comorbidities like aHUS.

### 2.1. Learning/Take-Home Points

1. Immunocompromised patients infected with COVID-19 can have an atypical, prolonged, and progressive presentation of their acute symptoms. It can take up to several weeks from the onset of initial symptoms until progression to respiratory failure or ARDS. This prolonged course is likely secondary to their impaired immune capacity to clear the virus and prevent replication
2. Complications of severe COVID-19 pneumonia encountered in this series have been variable, including secondary fungal infections, pneumothorax, pneumomediastinum, pulmonary embolism, pulmonary infarction, and atypical hemolytic-uremic syndrome
3. Our case series suggest the potential benefit of early management with hydrocortisone and remdesivir in this group of patients as evidenced by decreased COVID-19 viral loads from tracheal aspirates/BAL, resolved hypoxemia, and subsequently survival of all patients who received this concurrent combination management. It also highlights the importance of high clinical suspicion for and early management of suspected bacterial and fungal secondary infections with broad antibacterial and antifungal coverage, including mucormycosis, in preventing mortality

## Figures and Tables

**Table 1:** Summary of cases.

Cases	Age	Cause of immunosuppression	Duration of persistent symptoms (from initial diagnosis with COVID-19 until admission with acute respiratory failure)	Associated complications	Management	Clinical outcome
Case 1	16-year-old	H/o ALL s/p peripheral stem cell transplantation	3-4 weeks	Diffuse alveolar hemorrhage Atypical hemolytic-uremic syndrome Pulmonary infarction secondary to invasive mucormycosis	Hydrocortisone Eculizumab Vancomycin Cefepime Amphotericin (after pulmonary infarction)	Death

Case 2	12-year-old	H/o ALL s/p bone marrow transplant	4 weeks	Diffuse alveolar hemorrhage Pulmonary embolism	Remdesivir Hydrocortisone Tocilizumab Vancomycin Cefepime Amphotericin	Discharge home on room air

Case 3	14-year-old	P-ANCA vasculitis with end-stage renal disease	5 weeks	Diffuse alveolar hemorrhage Pneumothorax Pneumomediastinum Hyperferritinemia	Remdesivir Hydrocortisone Anakinra IVIG Plasmapheresis Vancomycin Cefepime Amphotericin	Discharge home on room air

Case 4	19-year-old	Granulomatosis with polyangiitis	5 weeks	Diffuse alveolar hemorrhage	Remdesivir Hydrocortisone Unasyn	Discharge home on room air

## Data Availability

The data that supports the findings of this study are available in the supporting information of this article.
